# Anxiety and depression symptoms among caregivers of care-recipients with subjective cognitive decline and cognitive impairment

**DOI:** 10.1186/s12883-016-0712-2

**Published:** 2016-10-03

**Authors:** Xiaoniu Liang, Qihao Guo, Jianfeng Luo, Fang Li, Ding Ding, Qianhua Zhao, Zhen Hong

**Affiliations:** 1 Department of Biostatistics, School of Public Health, Fudan University, Shanghai, 200032 China; 2 Institute of Neurology, Huashan Hospital, Fudan University, WHO Collaborating Center for Research and Training in Neurosciences, Shanghai, 200040 China; 3 School of Public Health and Key Laboratory of Public Health Safety, Fudan University, Shanghai, 200032 China; 4 Key Laboratory of Health Technology Assessment (Ministry of Health), Fudan University, Shanghai, 200032 China; 5 Department of Gerontology, Fuxing Hospital, Capital Medical University, Beijing, 100038 China

**Keywords:** Anxiety, Depression, HADS, Caregiver, Subjective cognitive decline, Risk factors

## Abstract

**Background:**

Caregivers of care-recipients with mild cognitive impairment (MCI) or dementia experience high caregiver burden; however, the psychiatric burden of caregivers of care-recipients with subjective cognitive decline (SCD) has not been investigated. We aimed to explore the prevalence of and risk factors for anxiety and depression symptoms among the caregivers of care-recipients with SCD and cognitive impairment.

**Methods:**

The Hospital Anxiety and Depression Scale (HADS) was used to examine the anxiety and depression symptoms among the caregivers of 343 care-recipients (84 with SCD, 120 with MCI and 139 with dementia) treated at the Memory Clinic of Huashan Hospital in Shanghai, China from May 2012 to October 2014. A logistic regression was used to explore the factors associated with caregiver’s anxiety and depression symptoms.

**Results:**

In total, 26.5 % of caregivers had anxiety symptoms, and 22.4 % had depression symptoms. Totals of 17.9, 30.0 and 28.8 % of caregivers of care-recipients with SCD, MCI or dementia, respectively, had anxiety symptoms (*P* = 0.1140), whereas 22.6, 24.2 and 20.9 %, respectively, had depression symptoms (*P* = 0.8165). The risk factors for caregiver’s anxiety symptoms were increased caregiver age as well as having care-recipients who were male, had higher Cohen Mansfield Agitation Inventory (CMAI) scores, and higher Geriatric Depression Scale (GDS) scores. The risk factors for caregiver’s depression symptoms were increased caregiver age as well as caring for care-recipients with MCI or SCD, those with lower Toronto Empathy Questionnaire (TEQ) scores, and those with higher GDS scores.

**Conclusions:**

Caregivers of care-recipients with SCD showed the same level of depression symptoms as those of care-recipients with MCI. Caregiver’s depression and anxiety symptoms were associated with their care-recipients’ psychiatric and behavioral syndromes.

## Background

Caregiver burden is common in dementia. This condition is not only associated with adverse emotional states and psychiatric morbidity but also poorer physical condition and worse financial and social consequences. Factors in three other domains are also relevant: care-recipient characteristics, caregiver characteristics and living conditions [1–3]. Previous studies have reported that approximately 22 % of caregivers experience clinical depression [4], and approximately 75 % of caregivers experience significant depression and anxiety symptoms [5, 6]. Mild cognitive deficits are not sufficient for a diagnosis of dementia; however, care-recipients with mild cognitive impairment (MCI) have a 10–12 % annual probability of progression to dementia [7–11]. One study also indicated that caregivers of care-recipients with MCI showed anxiety and depression symptoms [12].

Subjective cognitive decline (SCD) in older adults is increasingly recognized as a potential indicator of non-normative cognitive decline, and some people with this condition eventually progress to dementia [13–17]. The core of the definition of SCD is self-experienced concerns about persistent decline in one or more aspects of cognitive function that are typically informant-corroborated; however, they show no impairment in activities of daily living and typically scores within normal limits on standard neuropsychological tests [18–20]. A previous study showed that the annual conversion rate (ACR) of SCD to dementia is 2.33 % (95 % CI: 1.93–2.78 %), and that of SCD to MCI is 6.67 % (95 % CI: 4.70–8.95 %) [17].

Regarding the persistent cognitive decline and the high rate of its conversion, we assumed that both care-recipients and their caregivers would suffer from cognitive complaints. However, psychiatric symptoms among SCD-caregivers have been under-researched. This study explored the prevalence of and risk factors for anxiety and depression symptoms among caregivers of care-recipients with SCD and cognitive impairment using data from the Memory Clinic of Huashan Hospital in Shanghai.

## Methods

### Design and participants

A hospital-based cross-sectional survey of 343 pairs of care-recipients and their caregivers was conducted at the Memory Clinic of Huashan Hospital in Shanghai from May 2012 to October 2014. A caregiver was defined as someone who spent at least 8 h per week caring for a care-recipient, regardless of whether they lived together. The inclusion criteria were: the care-recipient was diagnosed with mild or moderate dementia, MCI or SCD; one primary caregiver cared for one care-recipient; the principal family caregiver was over 18 years old and took care of or lived with the care-recipient; the caregiver’s relationship with the care-recipient and their caring time were also considered when recruiting participants, therefore, spouses, daughters, sons, daughters-in-law and other relatives were included, regardless of whether they lived with their care-recipients. Those who simultaneously provided care for another family member with a chronic medical condition were excluded.

### Demographic factors and medical history

Demographic characteristics, including age, gender and education, were collected from the care-recipients and their caregivers. Additional information was collected regarding the caregiver such as their working status, experience in caring for care-recipients with dementia, familiarity with the care-recipient and their relationship with the care-recipient.

### Neurological, psychiatric and neuropsychological assessments

For each care-recipient, cognitive function scales covering domains of global cognition (i.e., executive function, visuospatial ability, memory, language and attention) were administered, including the Mini Mental State Examination (MMSE) [21], the Memory and Executive Screening (MES) [22], the Montreal Cognitive Assessment (MOCA) [23], the Rey-Osterrieth Complex Figure Test (CFT) [24], the Stroop Color Word Test (CWT) [25], the Boston Naming Test (BNT) [26], the Auditory Verbal Learning Test (AVLT) [27], the Symbol Digit Modalities Test (SDMT) [28], the Event-Based Prospective Memory Test (EBPM) and the Time-Based Prospective Memory Test (TBPM) [29, 30], the Animal Verbal Fluency Test (VFT) [31],the Trail Making Test (TMT) [32], the Judgement of Line Orientation (JLO) [33], the Word Memory Test (WMT) [34],the Visual Object and Space Perception (VOSP) [35], and the Clinical Dementia Rating Scale (CDR) [36]. Furthermore, the Geriatric Depression Scale (GDS) [37] and the Zung Self-Rating Anxiety Scale (ZSAS) [38] were also administered for each care-recipient. For each caregiver, the 16-item version of the Informant Questionnaire on Cognitive Decline in the Elderly (IQCODE) [39], the Toronto Empathy Questionnaire (TEQ) [40], the Cohen-Mansfield Agitation Inventory (CMAI) [41] and the Apathy Evaluation Scale (AES) [42] were administered to evaluate the psychiatric and neuropsychological symptoms of care-recipients.

The Hospital Anxiety and Depression Scale (HADS) is a popular clinical and research instrument used to screen for anxiety and depression symptoms in both hospital and community settings [43, 44]. The HADS facilitates the early identification of both anxiety and depression symptoms simultaneously; furthermore, it provides a separate score for each domain. It is easy to score and simple to interpret. The Chinese version of the HADS is available and shows acceptable reliability and validity [45–47]. Therefore, we used the HADS to evaluate the anxiety and depression symptoms of each caregiver. The HADS is composed of 14 items, seven of which relate to anxiety symptoms and seven concern depression symptoms. Each item is coded from 0 to 3. Therefore, the total scores for anxiety symptoms and depression symptoms can both vary from 0 to 21, representing the presence and severity of the symptoms. We used the score of 8 to define “caseness” for both anxiety symptoms and depression symptoms [43, 48, 49]. 

Four experienced neurologists and one neuropsychologist (QG, DD, QZ, FL, and ZH) who work at top institutions of neurology in China (Huashan Hospital and Fuxing Hospital) reviewed the functional, medical, neurological, psychiatric, and neuropsychological data, and reached a consensus regarding the Diagnostic and Statistical Manual of Mental Disorders-IV (DSM-IV) criteria for dementia [50]. Only care-recipients who were not diagnosed with dementia were considered for a diagnosis of MCI based on the Petersen’s criteria [51]. The other care-recipients were diagnosed with SCD using a broad research criteria for pre-MCI SCD, which included two presentations: (1) the care-recipients had to report self-experienced persistent decline in cognition compared to previous statue and that was unrelated to an acute event; (2) care-recipients have normal age-, gender-, and education-adjusted performance on standardized cognitive tests used to classify MCI.

### Statistical analysis

Continuous variables were expressed as mean and standard deviation (SD), and categorical variables were expressed as frequencies (%). We divided all care-recipients and caregivers into four groups according to the caregiver’s performance on the HADS: with anxiety [anxiety(+)], without anxiety [anxiety(−)], with depression [depression(+)], and without depression [depression(−)]. The Wilcoxon rank sum test and the Kruskal-Wallis H rank sum test were used to compare continuous variables, whereas Pearson Chi-squared test was used to compare categorical variables. Backward logistic regression models were used to explore risk factors for anxiety and depression symptoms among caregivers. Risk factors were presented as odds ratio (OR) with 95% confidence interval (95 % CI). The possible risk factors we adjusted for care-recipients included their gender, age, education, diagnosis, and presence of stroke as well as their IQCODE, TEQ, CMAI, GDS, ZSAS and AES scores; those for caregivers included their gender, age, education level, working status, and experience in caring for care-recipients with dementia as well as their familiarity with the care-recipient and their relationship with the care-recipient. Two-tailed tests were used for all analyses at a significance level of *P* < 0.05. The data were analyzed using SAS 9.3 (SAS Institute Inc., Cary, NC, USA).

## Results

The demographic, medical history, neurology tests, psychiatric assessment and neuropsychological assessment data of the 343 pairs of care-recipients and caregivers are shown below.

### Comparison of caregiver characteristics between caregiver groups with or without anxiety and depression

Totals of 17.9, 30.0 and 28.8 % of caregivers of care-recipients with SCD, MCI or dementia had anxiety symptoms (*P* = 0.1140), respectively; 22.6, 24.2 and 20.9 % of these caregivers had depression symptoms (*P* = 0.8165), respectively. Although caregivers of care-recipients with SCD had relatively low anxiety symptoms and relatively high depression symptoms, anxiety or depression symptoms were not associated with cognitive impairment severity (Table 1).

**Table 1:** Comparison of caregiver characteristics between caregiver groups with or without anxiety and depression

	All	Anxiety (+)	Anxiety (−)	*P*-value	Depression (+)	Depression (−)	*P*-value
Total prevalence, *n* (%)	343	91 (26.5)	252 (73.5)		77 (22.4)	266 (77.6)	
Prevalence of caregivers of care-recipients with, *n* (%)				0.1140			0.8165
SCD	84 (24.5)	15 (17.9)	69 (82.1)		19 (22.6)	65 (77.4)	
MCI	120 (35.0)	36 (30.0)	84 (70.0)		29 (24.2)	91 (75.8)	
Dementia	139 (40.5)	40 (28.8)	99 (71.2)		29 (20.9)	110 (79.14)	
Gender, male, *n* (%)	146 (43.7)	30 (34.5)	116 (47.0)	0.0436	33 (45.8)	113 (43.1)	0.6821
Age, years, mean(SD)	55.0 (16.4)	57.5 (15.1)	54.1(16.8)	0.1204	58.8 (14.9)	53.9 (16.7)	0.0290
Education level				0.0444			0.0394
Illiteracy, *n* (%)	49 (14.7)	16 (32.7)	33 (67.4)		14 (28.6)	35 (71.4)	
Primary, *n* (%)	73 (21.9)	18 (24.7)	55 (75.3)		18 (24.7)	55 (75.3)	
Middle, *n* (%)	154 (46.3)	43 (27.9)	111 (72.1)		30 (19.5)	124 (80.5)	
Junior and college, *n* (%)	15 (4.5)	5 (33.3)	10 (66.7)		5 (33.3)	10 (66.7)	
University and higher, *n* (%)	42 (12.6)	4 (9.5)	38 (90.5)		4 (9.5)	38 (90.5)	
Relationship with care-recipient				0.6361			0.3128
Spouse, *n* (%)	176 (52.1)	47 (26.7)	129 (73.3)		43 (24.4)	133 (75.6)	
Child, *n* (%)	141 (41.7)	35 (24.8)	106 (75.2)		26 (18.4)	115 (81.6)	
Other relative, *n* (%)	21 (6.2)	5 (23.8)	16 (76.2)		4 (19.0)	17 (81.0)	
Experience, *n* (%)	29 (9.2)	5 (6.1)	24 (10.3)	0.2616	8 (12.3)	21 (8.4)	0.3266
Work, *n* (%)	175 (52.9)	40 (46.0)	135 (55.3)	0.1336	34 (47.2)	141 (54.4)	0.2778
Familiarity with the care-recipient				0.3732			0.6333
Know well, *n* (%)	185 (58.0)	45 (24.3)	140 (75.7)		39 (21.1)	146 (78.9)	
Partially know, *n* (%)	120 (37.6)	38 (31.7)	82 (68.3)		29 (24.2)	91 (75.8)	
Know little, *n* (%)	14 (4.4)	3 (21.4)	11 (78.6)		3 (21.4)	11 (78.6)	

*Abbreviation*: *SD*: standard deviation; *SCD*: subjective cognitive decline; *MCI*: mild cognitive impairment. *P*-values concern the between-group comparisons of caregivers based on whether they are anxious or depressed

Table 1 also shows that 146 (43.7 %) caregivers were men, and the mean age was 55.0 (SD 16.4) years. The education levels of the caregivers included illiteracy (49, 14.7 %), primary education (73, 21.9 %), middle education (154, 46.3 %), junior and college education (15, 4.5 %), and university and higher education (42, 12.6 %). The following caregiver characteristics significantly differed: gender and education level regarding anxiety symptoms, and education level and age regarding depression symptoms. When caregivers were women or had low education levels, they were more likely to have anxiety symptoms; when caregivers were older or had low education levels, they were more likely to have depression symptoms.

### Comparison of care-recipient characteristics between caregiver groups with or without anxiety and depression

A total of 84 (24.5 %) care-recipients had SCD, 120 (35.0 %) had MCI, and 139 (40.5 %) had mild to moderate dementia (Table 1). Their mean age was 66.5 (SD 10.6) years. Moreover, 53.9 % were men, and their mean years of education were 8.9 (SD 3.6) years. The IQCODE, TEQ, CMAI, AES, ZSAS and GDS scores of care-recipients significantly differed between the two groups of caregivers with or without anxiety symptoms. Compared with the care-recipients of caregivers without anxiety symptoms, those of caregivers with anxiety symptoms had higher IQCODE scores (55.6 vs 50.8), lower TEQ scores (38.0 vs 41.8), higher CMAI scores (41.5 vs 35.4), lower AES scores (27.1 vs 31.6), higher ZSAS scores (36.9 vs 34.7) and higher GDS scores (6.5 vs 4.7s). The TEQ, CMAI, AES and GDS scores of care-recipients significantly differed between the groups of caregivers with or without depression symptoms. Compared with the care-recipients of caregivers without depression symptoms, those of caregivers with depression symptoms had lower TEQ scores (38.1 vs 41.5), higher CMAI scores (39.3 vs 36.3), lower AES scores (26.6 vs 31.5) and higher GDS scores (6.1 vs 4.9) (Table 2).

**Table 2:** Comparison of care-recipient characteristics between caregiver groups with or without anxiety and depression

	All	Anxiety (+)	Anxiety (−)	*P*-value	Depression (+)	Depression (−)	*P*-value
Gender, male, *n* (%)	185 (53.9)	52 (57.1)	133 (52.8)	0.4740	45 (58. 4)	140 (52.6)	0.3677
Age, years, mean (SD)	66.5 (10.6)	67.0 (10.2)	66.3 (10.8)	0.5487	66.1 (11.0)	66.6 (10.5)	0.9714
Education, years, mean (SD)	8.9 (3.6)	9.2 (3.6)	8.9 (3.6)	0.4228	9.1 (3.6)	8.9 (3.6)	0.4911
Stroke, *n* (%)	59 (17.3)	19 (20.9)	40 (15.9)	0.2850	16 (20.8)	43 (16.2)	0.3520
MMSE scores, mean (SD)	23.5 (5.1)	23.0 (5.5)	23.6 (4.9)	0.4748	23.4 (5.3)	23.5 (5.0)	0.8552
MES scores, mean (SD)	64.2 (22.0)	62.4 (21.9)	64.8 (22.0)	0.3024	61.2 (23.8)	65.1 (21.4)	0.2618
IQCODE scores, mean (SD)	52.0 (15.3)	55.6 (14.0)	50.8 (15.6)	0.0120	53.6 (17.2)	51.6 (14.8)	0.1663
TEQ scores, mean (SD)	40.8 (8.8)	38.0 (8.0)	41.8 (8.9)	< 0.0008	38.1 (8.0)	41.5 (8.9)	0.0041
CMAI scores, mean (SD)	37.0 (10.1)	41.5 (12.1)	35.4 (8.8)	< 0.0001	39.3 (11.1)	36.3 (9.8)	0.0135
AES scores, mean (SD)	30.4 (13.4)	27.1 (13.1)	31.6 (13.4)	0.0078	26.6 (13.6)	31.5 (13.2)	0.0077
ZSAS scores, mean (SD)	35.2 (7.2)	36.9 (7.8)	34.7 (6.9)	0.0212	35.5 (7.4)	35.2 (7.2)	0.7837
GDS scores, mean (SD)	5.2 (3.7)	6.5 (3.9)	4.7 (3.6)	0.0003	6.1 (3.7)	4.9 (3.7)	0.0138

*Abbreviation*: *SD* standard deviation, *MMSE* Mini Mental State Examination, *MES* Memory and Executive Screening; *IQCODE* Informant Questionnaire on Cognitive Decline in the Elderly, *TEQ* Toronto Empathy Questionnaire, *CMAI* Cohen-Mansfield Agitation Inventory, *AES* Apathy Evaluation Scale, *ZSAS* Zung Self-Rating Anxiety Scale, *GDS* Geriatric Depression Scale. *P*-values concern the between-group comparisons of caregivers based on whether they are anxious or depressed

### Possible risk factors for caregiver’s anxiety and depression symptoms

After adjusting the possible risk factors (gender, age, education, presence of stroke, and diagnosis as well as the IQCODE, TEQ, CMAI, GDS, ZSAS and AES scores for care-recipients; and gender, age, education level, working status, experience caring for care-recipients with dementia, familiarity with the care-recipient and relationship with the care-recipient for caregivers), we found that possible risk factors for caregiver’s anxiety symptoms were having a male care-recipient (OR = 2.19, 95 % CI:1.07–4.48), a higher CAMI score (OR = 1.06, 95 % CI: 1.02–1.10), a higher GDS score (OR = 1.15, 95 % CI:1.05–1.27) and increased caregiver age (OR = 1.02, 95 % CI:1.00–1.05). The risk factors for caregiver’s depression symptoms were having a lower TEQ score (OR = 1.07, 95 % CI:1.02–1.11), a care-recipient with MCI(OR = 2.76, 95 % CI:1.11–6.88) or SCD(OR = 3.40, 95 % CI:1.16–10.01) compared with one with dementia, a higher GDS score (OR = 1.13, 95 % CI:1.03–1.24) and increased caregiver age (OR = 1.02, 95 % CI:1.00–1.05) (Table 3).

**Table 3:** Possible risk factors for caregiver’s anxiety and depression symptoms

Anxiety (+)		Depression (+)	
Variables	OR (95 % CI)	Variables	OR (95 % CI)
Male care-recipient vs Female care-recipient	2.19 (1.07,4.48)	SCD vs MCI MCI vs dementia SCD vs dementia	1.23 (0.49,3.12) 2.76 (1.11,6.88) 3.40 (1.16,10.01)
CMAI (scores, increasing)	1.06 (1.02,1.10)	TEQ (scores, decreasing)	1.07 (1.02,1.11)
GDS (scores, increasing)	1.15 (1.05,1.27)	GDS (scores, increasing)	1.13 (1.03,1.24)
Age of caregivers (years, increasing)	1.02 (1.00,1.05)	Age of caregivers (years, increasing)	1.02 (1.00,1.05)

*Notes*: Multivariate logistic regression model adjusted for the gender, age, education, presence of stroke, and diagnosis as well as the IQCODE, TEQ, CMAI, GDS, ZSAS and AES scores of care-recipients; the model also adjusted for the gender, age, education level, and working status of caregivers as well as their experience caring for care-recipients with dementia, familiarity with the care-recipient and relationship with the care-recipient

*Abbreviations*: *OR* odds ratio, *CI* confidence interval, *MCI* mild cognitive impairment, *SCD* subjective cognitive decline, *CMAI* Cohen-Mansfield Agitation Inventory, *GDS* Geriatric Depression Scale, *TEQ* Toronto Empathy Questionnaire

## Discussion

In our study, 26.5 % of caregivers had anxiety symptoms, and 22.4 % had depression symptoms. For caregivers of care-recipients with dementia, MCI or SCD, the rates of anxiety symptoms in were 28.8, 30.0 or 17.9 %, respectively; whereas the rates of depression symptoms were 20.9, 24.2 or 22.6 %, respectively. The risk factors for caregiver’s anxiety symptoms were higher CMAI scores, higher GDS scores, increased caregiver age and having a male care-recipient. The risk factors for caregiver’s depression symptoms were lower TEQ scores, higher GDS scores, increased caregiver age and caring for care-recipients with SCD or MCI.

The prevalence of depression symptoms among caregivers of care-recipients with SCD or MCI was higher than that among caregivers of care-recipients with dementia. The prevalence of anxiety and depression symptoms among caregivers of care-recipients with dementia was in accordance with that in previous studies [4, 6, 52]. The pooled depression rate of caregivers of care-recipients with MCI (CESD scores ≥ 16 or equivalent, total *N* = 929) was 23 % (11–24.6 % for individual studies) [12], which is in accordance with our results. One study suggested that dementia plays a significant role in caregiver’s depression among Latino families compared with cognitive impairment but not dementia (CIND) [53]. However, most studies have found that depression and anxiety symptoms among caregivers of care-recipients with dementia are not associated with level of cognitive impairment [54–56]. Another study showed that cognitive function and dementia severity were not correlated with caregiver burden [57]. One possible explanation for our findings is that care-recipients with SCD might often complain about their own declining memory and refer to the effect of their declining memory on activities of daily living. These complaints draw the attention of caregivers and require support and care. When caregivers hear the complaint about declining memory from care-recipients who didn’t previously complain, and are unable to access the support that care-recipients need, caregivers might feel depressed. Another possible explanation is that prior to diagnosis, psychiatric symptoms are more stressful to caregivers because their etiology is unclear, leading to higher caregiver’s depression. After diagnosis, psychiatric symptoms are likely attributed to the process of MCI. With the care-recipient’ further cognitive decline, however, the caregivers become familiar with the disease, able to care for the care-recipients and able to accept the current situation. A third reason might be that care-recipients with mild or moderate dementia, not the severe dementia observed in our study, have relatively mild mental symptoms and do not become a heavy burden on their caregivers.

We found that care-recipient GDS scores were a risk factor for both anxiety and depression symptoms among caregivers; however, we did not find that care-recipient ZSAS or AES scores were associated with caregiver’s anxiety or depression symptoms. Most studies have found that depression and anxiety symptoms in caregivers of people with dementia are strongly associated with patients’ psychiatric symptoms, particularly, depression symptoms [55, 58–62]. Furthermore, we found that older caregivers had higher risks of both anxiety and depression symptoms. In an Italian multicenter study of 419 elderly outpatients with dementia and their caregivers, increased caregiver age was a major risk factor for both depression and anxiety (BDDA) among caregivers [63]. However, another study of caregivers of people with cognitive impairment did not find that age was an important predictor of caregiver’s anxiety [6, 64]. This finding might be because the physical functions of older caregivers are worse; thus, they become anxious and depressed given the increased burden.

Our study showed that care-recipient CMAI scores were only related to caregiver’s anxiety symptoms. A study of 35 patient-caregiver pairs evaluated at two university-affiliated dementia clinics found highly significant correlations between patient agitation and caregiver burden (*r* = 0.59, *p* = 0.0002) as well as between depression and caregiver’s depression (*r* = 61, *p* = 0.0001) [65]. A Chinese study found a positively correlation between the agitation among patients with dementia in nursing homes and the stress of 40 nursing staff members [66]. The reason for this finding might be that in addition to the constant daily care they provide, nursing staff must also spend more energy and time preventing care-recipient' self-harm and harm to others. If these effects cannot be predicted or prevented, then it might lead to serious consequences, and make caregivers feel persistently anxious. Furthermore, our study also showed that caregivers of males were more likely to suffer from anxiety symptoms, unlike previous studies. A previous study [61] found that female caregivers were more likely to experience psychological distress. The LASER-AD study found that being a female caregiver predicted having an anxiety disorder [67]. Our result might be because males are the core members of Chinese families; however, they are more impulsive and harder to control. Caregivers must take on more responsibilities (e.g., physical or financial problems) than before, which might result in caregiver’s anxiety symptoms.

We also found that care-recipient TEQ scores were a risk factor for caregiver’s depression symptoms. Previous studies have not investigated the relationship between empathy and depression symptoms. One possible reason might be that cognitive impairment is difficult to treat with either behavioral or pharmacologic methods, leading to embarrassment among care-recipients, which might contribute to caregivers’ feelings of social isolation.

In our study, some characteristics of caregiver (i.e., gender, education level, the caregiver’s working status, experience in caring for care-recipients with dementia, familiarity with the care-recipient and their relationship with the care-recipient) were not associated with caregiver’s anxiety and depression symptoms. Having less education was significantly associated with depression of caregivers of care-recipients with MCI in two [68, 69] of three studies [68–70]. Caregiver gender [70–72] and relationship with care-recipients [69, 70] did not predict depression of caregivers of care-recipients with MCI. Having less dementia knowledge significantly predicted depression of caregivers of care-recipients with MCI [70]. Depression of caregivers of care-recipients with dementia or cognitive impairment without dementia was associated with whether the caregiver was the care-recipient’s spouse, and whether the care-recipient had dementia or CIND [73]. The reason may be that we studied the anxiety and depression symptoms among caregivers of care-recipients with subjective cognitive decline and cognitive impairment, not only with cognitive function impairment.

To our knowledge, this study is the first to report the prevalence of and risk factors for anxiety and depression symptoms among the caregivers of care-recipients with SCD. The first limitation of our study is that the cross-sectional design limits any conclusions in a causal or predictive direction. Future work should examine these relationships longitudinally to more fully characterize the direction of cause and effect. Second, we adjusted for as many risk factors as possible in the logistic regression model; however, we can’t exclude the possible influence of uncollected risk factors such as the specific care time of caregivers, alcohol drinking habits, disease history (e.g., like head injury, coronary heart disease, hypertension and diabetes), economic status, physical and psychological illness among caregivers, and drugs that affect mental health. Third, there are only care-recipients with mild or moderate dementia, not the severe dementia in our study, but care-recipients with the severe dementia may cause serious impacts on their caregivers.

## Conclusions

Our study suggests that clinicians should be aware of high rates of anxiety and depression symptoms among caregivers of care-recipients with SCD or MCI. We should also attend to the risk factors for anxiety and depression symptoms among caregivers such as care-recipient depressions, empathy and agitation. Furthermore, many caregivers require more social support via training as well as physical and mental health care. Such programs should provide caregivers with support to address their burden and educate them learn about cognitive impairment.

## Abbreviations

AES: Apathy evaluation scale
AVLT: Auditory verbal learning test
BDDA: Burden, distress, depression and anxiety
BNT: Boston naming test
CDR: Clinical dementia rating scale
CESD: Epidemiologic studies depression scale
CFT: Rey-Osterrieth complex figure test
CI: Confidence interval
CMAI: Cohen-Mansfield agitation inventory
CWT: Stroop color word test
EBPM: Event-based prospective memory test
GDS: Geriatric depression scale
HADS: The hospital anxiety and depression scale
IQCODE: Informant questionnaire on cognitive decline in the elderly
JLO: Judgement of line orientation
MCI: Mild cognitive impairment
MES: Memory and executive screening
MMSE: Mini mental state examination
MoCA: Montreal cognitive assessment
OR: Odds ratio
SCD: Subjective cognitive decline
SD: Standard deviation
SDMT: Symbol digit modalities test
TBPM: Time-based prospective memory test
TEQ: Toronto empathy questionnaire
TEQ: Toronto empathy questionnaire
TMT: Trail making test
VFT: Animal verbal fluency test
VOSP: Visual object and space perception
WMT: Word memory test
ZSAS: Zung self-rating anxiety scale
